# In silico analysis of epitope-based CadF vaccine design against *Campylobacterjejuni*

**DOI:** 10.1186/s13104-020-05364-z

**Published:** 2020-11-10

**Authors:** Mona Moballegh Naseri, Saeed Shams, Mohammad Moballegh Naseri, Bita Bakhshi

**Affiliations:** 1 Cellular and Molecular Research Center, Qom University of Medical Sciences, 3736175513 Qom, Iran; 2 Department of Computer and IT, Shahab-Danesh University, Qom, Iran; 3 Department of Bacteriology, Faculty of Medical Sciences, Tarbiat Modares University, Tehran, Iran

**Keywords:** *Campylobacter* *jejuni*, Vaccine, CadF, In Silico, Bioinformatics, Epitope

## Abstract

**Objective:**

Vaccination is an important strategy for the eradication of infectious diseases. CadF protein of* Campylobacter jejuni* is one of the important factors in the pathogenesis of this bacterium. The purpose of this work was to perform a bioinformatics study to identify an epitope-based CadF vaccine, as a subunit vaccine. Full protein sequences of CadF were extracted from the NCBI and UniProt databases and subjected to in silico evaluations, including sequence analysis, allergenicity, antigenicity, epitope conservancy, and molecular docking assessments done by different servers.

**Results:**

The results showed that CadF was a highly conserved protein belonging to the outer member proteins superfamily. Among the evaluated epitopes, LSDSLALRL was identified as an antigenic and non-allergenic peptide with a suitable structure for vaccine development. It was also able to stimulate both T and B cells. This 9-mer peptide was located in 136–144 segment of CadF protein and interacted with both HLA-A 0101 and HLA-DRB1 0101 alleles. Overall, the obtained theoretical results showed that CadF protein could be used for designing and evaluating a new effective vaccine against *C. jejuni*.

## Introduction

*Campylobacter jejuni* (*C. jejuni*) is one of the significant pathogens belonging to the genus *Campylobacter*. The bacterium is a Gram-negative, curved, flagellated, and rod-shaped pathogen which can be transmitted to humans through direct contact with animals and consumption of contaminated food, water, and unpasteurized milk [1, 2]. A gastrointestinal problem commonly caused by *C. jejuni*, especially in children, is called campylobacteriosis [3].

Vaccination is unanimously accepted as an appropriate strategy to eradicate and prevent infectious diseases [4]. Among different types of vaccines, subunit vaccines usually contain parts of the target microorganisms and are known to be safe and effective vaccines for humans [5]. These vaccines activate both humoral- and cell-mediated immune mechanisms to protect them against pathogens. To obtain novel epitope-based subunit vaccines, the identification and prediction of antigenic epitopes by bioinformatics tools are recommended. These provide new theoretical approaches for the design of the vaccines based on immunological databases [6–11].

CadF (*Campylobacter* adhesion to fibronectin) as one of the virulence factors of *C. jejuni* is a conserved, genus-specific, and 37 kDa outer membrane protein that binds to fibronectin and facilitates bacterial colonization of host cells. The protein is also identified as an antigen and could induce massive immune responses [6, 12, 13].

Although there are some studies on the development of vaccine candidates based on the subunit vaccines of *C. jejuni* [14, 15], little is known about CadF potential to be independently considered in the expansion of a protective vaccine.

This study aimed to analysis CadF protein in order to identify epitope-based peptide candidates and evaluate its proteomic database using bioinformatics tools and servers for developing a new vaccine candidate. Therefore, the work was solely an "in silico" study.

## Main text

### Methods

#### Protein sequences evaluation

CadF protein sequences were obtained from both NCBI (Accession numbers: AGI56319.1, AAD28174.1, CAL35585.1, CAL35585.1, QFZ66584.1, QMX64581.1, EEA6200125.1, WP_004314268.1, EEO9446683.1, ACP52761.1) (https://www.ncbi.nlm.nih.gov/protein) and UniProt databases (Accession numbers: Q0P8D9, A0A649PTZ6, A0A430SVE4, A0A5T0DIN6, A0A5T1CIC0, A0A6H8RR06) (https://www.uniprot.org) in FASTA format. Evolutionary analysis was performed by multiple alignment and phylogenetic tree of the sequences using the ClustalW2 (https://www.ebi.ac.uk/Tools/msa/clustalw2) tool and Molecular Evolutionary Genetics Analysis software Version 7 (MEGA 7).

#### Protein characterization

The three-dimensional structures and biological functions of the protein were recognized by Phyre2 (www.sbg.bio.ic.ac.uk/phyre2) as an online protein fold recognition server. The protein structures were also analyzed by the PSIPRED (https://bioinf.cs.ucl.ac.uk/psipred) server.

Two TMHMM (https://www.cbs.dtu.dk/services/TMHMM) and ProtParam (https://web.expasy.org/protparam) servers were used to predict exo-membrane amino acid sequences and physico-biochemical characteristics of CadF protein.

#### Allergenicity and antigenicity assessment

The AllerTOP (www.ddg-pharmfac.net/AllerTOP) and AllergenFP (ddg-pharmfac.net/AllergenFP) web servers were used to determine the allergenicity of CadF protein and common peptides. The AllergenFP was databased to obtain a set of options for predicting allergens. The VaxiJen (https://www.ddg-pharmfac.net/vaxijen/VaxiJen/VaxiJen.html) server was also used to forecast the antigenicity of the sequences.

#### Epitope conservancy assessment

The MHC I and MHC II (Major Histocompatibility Complex) epitopes were analyzed by the Immune Epitope Database and Analysis Resource or IEDB (https://www.iedb.org/), NetCTL (www.cbs.dtu.dk/services/NetCTL), NetMHC (www.cbs.dtu.dk/services/NetMHC), NHLApred (https://crdd.osdd.net/raghava/nhlapred/), SYFPEITHI (www.syfpeithi.de), and MHC2Pred (https://crdd.osdd.net/raghava/mhc2pred/) online servers.

The B cell epitopes were identified using the IEDB, SVMTriP (https://sysbio.unl.edu/SVMTriP/), and BCPREDS (https://ailab.ist.psu.edu/bcpred/) servers by setting a default specificity of 75%; the threshold value of 0.5 was considered for ABCpred (https://crdd.osdd.net/raghava/abcpred) server. Linear and discontinuous B cell epitopes were also predicted by the BepiPred (https://www.cbs.dtu.dk/services/BepiPred) server. This server was applied to predict B cell epitopes through the combination of a Hidden Markov Model (HMM) and a propensity scale method. Each epitope identified by these servers was checked to determine the allergenicity and antigenicity properties. The identified common epitopes were analyzed as predicted epitopes, and finally, the best common peptides were selected.

#### Molecular docking of adopted epitope and alleles

The three-dimensional structures of HLA-A 0101 and HLA-DRB1 0101 alleles were extracted from the Protein Data Bank (https://www.rcsb.org) with UniProt accession numbers Q5SUL5 and P01911, respectively. The PyMOL Molecular Graphics System (https://pymol.org) was used to analyze the three-dimensional structure of the best epitope. Final epitope and alleles were edited by Notepad^++^, and the interaction between them (epitope/ HLA-A 0101 allele of MHC I and epitope/ HLA-DRB1 0101 allele of MHC II) was assessed with the help of Molecular Virtual Docker and Molecular Virtual Viewer software. Finally, the interfaces between the epitope and alleles were selected based on a grid, computed on three axes, including x: − 0.16, y: − 17.63, and z: − 15.67.

## Results

### Analysis of CadF sequences

The complete sequences of CadF protein contained 319 amino acids, and multiple sequence alignment confirmed that this protein was a highly conserved protein among Campylobacter species. It was shown to belong to the outer member proteins superfamily (ompA), and an ompA-like domain was identified in the 193–287 position of the protein. The result of the phylogenic tree also confirmed CadF classification in the outer membrane proteins superfamily (data not shown).

### Characterization of CadF

Using the ProtParam server, the MV (molecular weight) and pI (isoelectric point) parameters were determined as 35,979.04 Da and 5.89, respectively. The aliphatic index was 69.12, and the GRAVY (grand average of hydropathicity) of the protein was − 0.679. As a result, the amino acids of CadF protein had hydrophobicity and acidity properties (pI ≤ 7.35). The aliphatic index included alanine, valine, isoleucine, and leucine amino acids, indicating the thermostability of the protein. Moreover, the TMHMM server data analysis results also confirmed that CadF was an outer membrane protein. According to the obtained result from the Phyre2 server, it was predicted that CadF was a stable target.

The PSIPRED server showed the graphical results of secondary structures of the protein, indicating a sheet, helix, and extracellular transmembrane structure. In addition, the Phyre2 server showed the three-dimensional structure of the modeled CadF with a 97% confidence score and 192 known-domain alignments. The structural content included 16% alpha-helix, 41% beta strands, and 16% disordered regions. Also, the prediction of CadF protein showed a binding site at glutamate-histidine-lysine residues and a large amount of metallic heterogenic sections in its structure.

### Evaluation of antigenicity and allergenicity

The score of antigenic prediction of CadF protein was calculated as ~ 0.79 by the VaxiJen server. The results showed that the protein was probably an antigen and could be used for further analysis. The AllergenFP server data indicated the highest Tanimoto similarity index of 0.82 for the protein; therefore, it could not be an allergen. The AllerTOP server data analysis results also confirmed the finding.

### Prediction of T and B cell epitopes

The best score for predicting T cell epitopes was selected from the SYFPEITH, IEDB, NetCTL, NHLAPred, and NetMHC servers. The epitopes of MHC I (A 0101, A 0201, and B 2705) and MHC II (DRB1 0101 and DRB1 0401) were the most frequent epitopes among Iranian alleles that were considered in this study. Using the Kolaskar & Tongaonkar Antigenicity method on the IEDB server, a graph was plotted, suggesting the yellow areas as B cell epitopes (Additional file 1: A). According to the obtained results, some epitopes such as VLFGADNNV, GLASVLFGA, LSDSLALRL, etc. were the most common epitopes among T and B cells. Further detailed information about the predicted MHC I, II, and B cell epitopes are presented in Tables 1a,b and 2, respectively. Overall, the results showed that the best epitope was LSDSLALRL located in 136–144 regions of CadF with antigenic properties and no allergenic specifications. The three-dimensional structure of the final epitope painted by the PyMOL is showed in Additional file 1: B. Therefore, it was suggested as a candidate vaccine for further analysis.

**Table 1:**
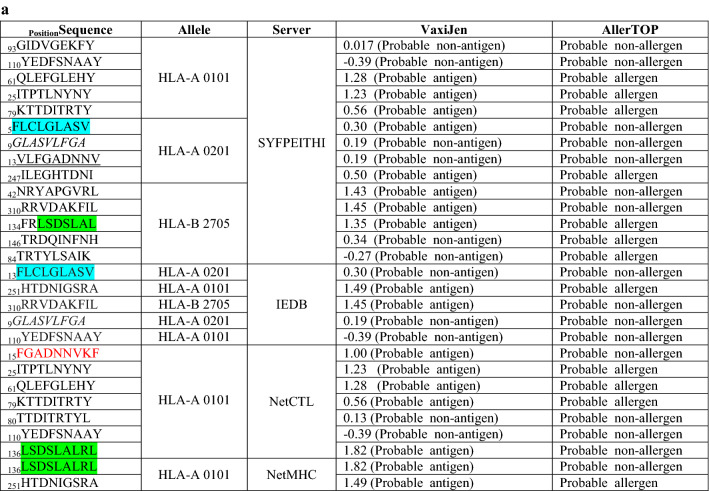

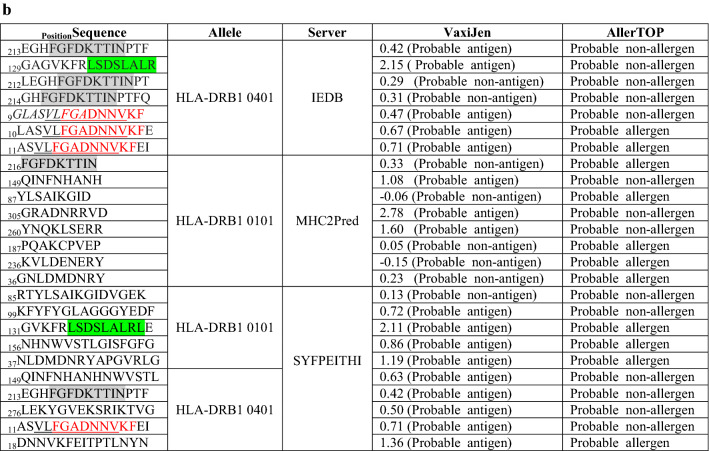
Predicted epitopes of MHC I (a) and MHC II (b) and their antigenicity and allergenicity properties. The most common peptides are marked in highlights

**Table 2:**
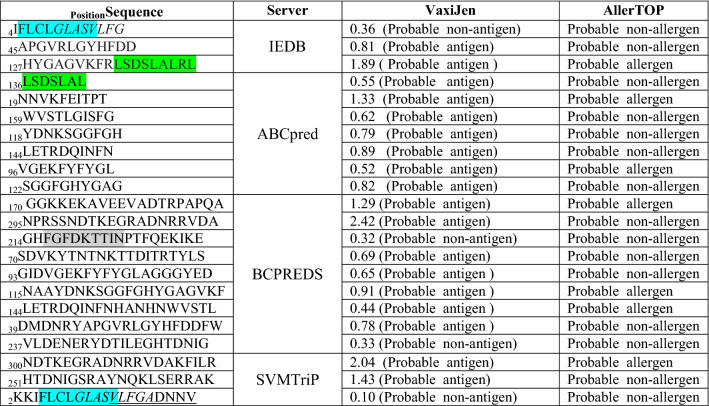
Predicted epitopes of B cell and their antigenicity and allergenicity properties. The most common peptides are marked in highlights

### Analysis of docking

The bindings of the best epitope to the desired HLA molecules were observed by Molecular Virtual Docker software, and five models were estimated. The proposed models showed the interaction of the epitope side chains with the cavities in the groove of MHC I and II (Additional file 1: C). The energies of the bonding models resulted from the binding of LSDSLALRL peptide to HLA-A 0101 consisted of − 26.18, − 18.62, − 12.77, and − 12.56 kcal/mol. The best scores of the peptide docking to HLA-DRB1 0101 were computed as − 109.86, − 99.52, − 98.40, and − 85.79 kcal/mol. According to the principles of docking energy evaluation, the model with the most negative docking results was selected as the best model with energies of − 26.18 and − 109.86 kcal/mol, which were related to HLA-A 0101 of MHC I and HLA-DRB1 0101 of MHC II, respectively.

## Discussion

This study was focused on the immunogenic protein CadF to design a hypothetical vaccine through bioinformatics tools. New candidate epitope-based vaccines identified using these tools could dramatically reduce the number of in vitro and in vivo tests [16]. The previous studies have reported some candidates to suggest an effective vaccine against *C. jejuni*. Despite many efforts to make a vaccine, no approved vaccine against *C. jejuni* has been developed as suitable so far [17, 18].

T and B cell epitopes were collected from different servers, and the best epitope was elicited to make an effective vaccine against *C. jejuni*. The present study showed the accurate topology model based on the Phyre2 server, predicting CadF as a stable target. This analysis helped design a novel hypothetical vaccine according to the sequence profile, spatial structure, and dimensions of the protein.

LSDSLALRL epitope was selected as the best potential vaccine candidate without any evidence of allergenicity. The epitope was located in 136–144 regions and could interact with HLA-A 0101 according to the results collected from many above-mentioned servers. In a study by Yasmin et al. (2016), gaining their knowledge of CadF protein based on just IEDB and SYFPEITHI servers, FRLSDSLAL epitope of the protein was suggested as a good choice for vaccine development [19].

It is clear that the epitope selected in this study is fairly matched (77.77%) with the epitope presented by Yasmin et al. (LSDSLALRL and FRLSDSLAL, which are marked by underline). This similarity could support the claim of suitability of the selected epitope for designing an effective vaccine against *C. jejuni*. Based on the AllerTOP server, the presented epitope by Yasmin et al. could probably be estimated as an allergen, while no allergenicity was observed for epitope "LSDSLALRL" in this study.

In addition, CadF is a significant protein for colonization, and maximum attachment could be detected in regions of the fibronectin-binding domain, including phenylalanine-arginine-leucine-serine (FRLS) residues of the protein [20]. Although only 50% of the selected epitope amino acids were identified as the binding site to host cells, multiple servers confirmed that this region had a high score for vaccine development.

According to the aliphatic index, alanine, valine, isoleucine, and leucine amino acids were detected in the protein structure, proposing it as a thermostable protein. These amino acids in thermophilic bacteria, e.g. *C. jejuni*, are significantly higher than that of ordinary proteins [21]. This proposes another advantage of CadF for the development of an effective vaccine. Heat stability is an important feature in vaccine production, which can simplify the logistics of vaccine distribution and expand the immunization coverage [22].

## Conclusion

It is suggested that CadF protein of *C. jejuni* could be used to prepare an effective vaccine for disease prevention. However, to predict an actual vaccine without any side effect, knowledge of the pathogenesis and molecular structure of *C. jejuni* needs to be improved through in vitro and in vivo studies in parallel with in silico research.

## Limitations

There were some limitations in the use of some servers. In addition, due to the limited funding and current facilities of our laboratory, it was not possible to validate the results through in vitro and in vivo projects**.**

## Supplementary information

**Additional file 1.** **A**: B cell epitopes of CadF protein; **B**: Three-dimensional structure of final epitope "LSDSLALRL"; **C**: Molecular docking analysis.

## Data Availability

The datasets used and/or analyzed during the current study are available from the corresponding author on reasonable request.

## Abbreviations

OmpA: Outer member proteins
CadF: *Campylobacter* Adhesion to Fibronectin
MHC: Major histocompatibility complex
MV: Molecular weight
pI: Isoelectric point
kcal/mol: Kilocalorie/mole
NCBI: National Center for Biotechnology Information
