# Supplementary material for: In silico analysis of epitope-based CadF vaccine design against Campylobacterjejuni
Source: BMC Res Notes. 2020 Nov 10;13:518. doi: 10.1186/s13104-020-05364-z (PMC7652678; doi:10.1186/s13104-020-05364-z)
Supplement: Supplementary file 1 — Additional file 1. A: B cell epitopes of CadF protein; B: Three-dimensional structure of final epitope "LSDSLALRL"; C: Molecular docking analysis. [file 13104_2020_5364_MOESM1_ESM.docx]

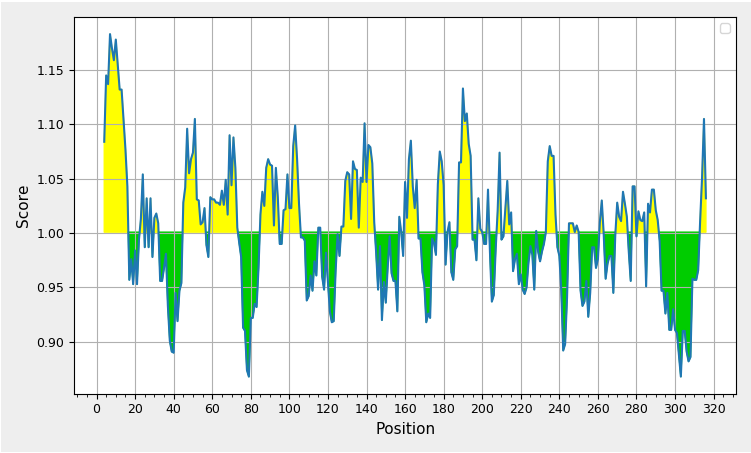


**A**. B cell epitopes of CadF protein. The estimated epitopes are indicated in yellow regions above the threshold line. Y and X axes depict residue scores and positions in amino acids, respectively.


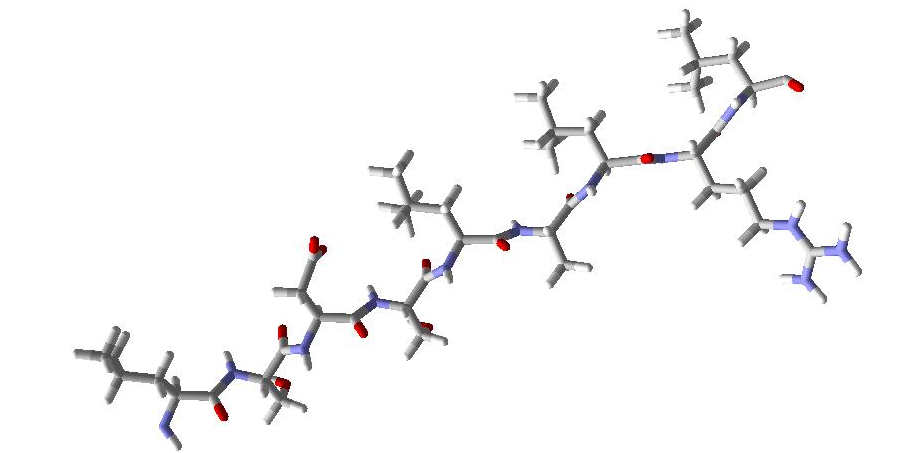


**B**. Three-dimensional structure of final epitope "LSDSLALRL ".

a:


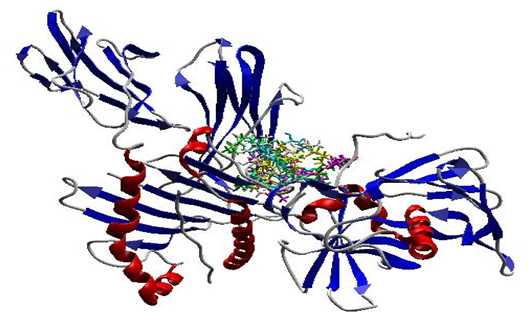


b:


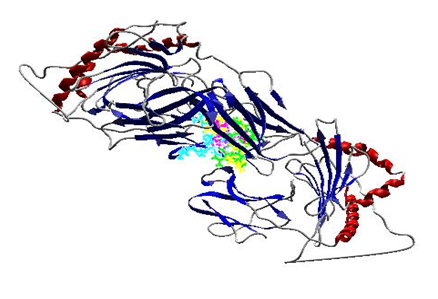


**C.** Molecular docking analysis. (a) HLA-A 0101 and LSDSLALRL. (b) HLA-DRB1 0101 and LSDSLALRL. The alleles are showed as secondary structures and epitopes are displayed as colorful sticks.
